# Effect of an orientation group for patients with chronic heart failure: randomized controlled trial ^1^

**DOI:** 10.1590/1518-8345.2167.2982

**Published:** 2018-01-08

**Authors:** Cristina Silva Arruda, Juliana de Melo Vellozo Pereira, Lyvia da Silva Figueiredo, Bruna dos Santos Scofano, Paula Vanessa Peclat Flores, Ana Carla Dantas Cavalcanti

**Affiliations:** 2 MSc, RN, Instituto Estadual de Cardiologia Aloysio de Castro, Rio de Janeiro, RJ, Brazil.; 3 Doctoral student, Universidade Federal Fluminense, Niterói, RJ, Brazil. RN, Hospital Universitário Clementino Fraga Filho, Universidade Federal do Rio de Janeiro, Rio de Janeiro, RJ, Brazil.; 4 Doctoral student, Universidade Federal Fluminense, Niterói, RJ, Brazil. Scholarship holder at Coordenação de Aperfeiçoamento de Pessoal de Nível Superior (CAPES), Brazil.; 5 RN.; 6 Doctoral student, Universidade Federal Fluminense, Niterói, RJ, Brazil. Assistant Professor, Universidade Federal Fluminense, Niterói, RJ, Brazil.; 7 PhD, Associate Professor, Universidade Federal Fluminense, Niterói, RJ, Brazil.

**Keywords:** Heart Failure, Nursing, Self-help Groups, Self Care, Patient Compliance, Clinical Trial

## Abstract

**Objective:**

To evaluate the effect of the orientation group on therapeutic adherence and self-care among patients with chronic heart failure.

**Method:**

Randomized controlled trial with 27 patients with chronic heart failure. The intervention group received nursing consultations and participated in group meetings with the multi-professional team. The control group only received nursing consultations in a period of four months. Questionnaires validated for use in Brazil were applied in the beginning and in the end of the study to assess self-care outcomes and adherence to treatment. Categorical variables were expressed through frequency and percentage distributions and the continuous variables through mean and standard deviation. The comparison between the initial and final scores of the intervention and control groups was done through the Student’s t-test.

**Results:**

The mean adherence in the intervention group was 13.9 ± 3.6 before the study and 4.8 ± 2.3 after the study. In the control group it was 14.2 ± 3.4 before the study and 14.7 ± 3.5 after the study. The self-care confidence score was lower after the intervention (p=0.01).

**Conclusion:**

The orientation group does not improve adherence to treatment and self-care management and maintenance and it may reduce confidence in self-care. Registry REBEC RBR-7r9f2m.

## Introduction

Despite the development of new technologies, scientific advances and the achievement of better social and economic conditions in recent decades, a high incidence of heart failure (HF) is still frequently observed in Brazil and in the world^1^. 

Limiting symptoms such as fatigue, dyspnea and angina are common in these patients and may be accompanied by memory loss and difficulty in concentration, making them incapable and restricting them in their activities of daily living^2^ ^-^ ^3^_._ This decrease in functional capacity may compromise adherence to treatment and self-care, leading to higher rates of hospitalization and death^4^.

A study found a low rate of self-care in 116 patients with HF and attributed this data to the rates of 44.8% patients with cognitive impairment and 52.6% with no nursing follow-up^5^.

Educational programs are an important tool to improve the management of self-care by the multi-professional team specialized in the follow-up of patients with HF^6^. However, it is difficult to identify the appropriate strategies and scenarios, since the interventions are heterogeneous, as well as the number of professionals involved, hindering the evaluation of results^5^. 

Thus, nurses working in specialized HF clinics seek strategies that can increase adherence to treatment and enhance self-care, improving quality of life and reducing hospital readmissions^5^ ^-^ ^7^. Group interventions are one of the strategies for disease management programs^8^ ^-^ ^11^. However, there are no studies that have proven their effectiveness in adherence to treatment and in self-care management, maintenance and confidence. 

An integrative review of 42 articles on interventions proposed and implemented by nurses to optimize self-care in patients with HF found that most of the studies were directed to patients, excluding the participation of caregivers and family members, and addressed three to four factors of self-care, such as diet, adherence to medication, daily weight, physical activity and monitoring of signs and symptoms of decompensated HF. There was a predominance of verbal instructions, but written materials and information technology such as CD-ROMs, DVDs and videos, as well as telehealth, were also used. Few studies discussed group activities^12^.

Orientation groups are used as a supplement to the guidelines provided during the outpatient visit. A study conducted in the Netherlands evaluated self-care and quality of life among 317 HF patients for 12 months after participating in 21 group sessions. The intervention showed improvements in cognitive management of symptoms (p<0.001), self-care behavior (p=0.008) and quality of life (p=0.005), but no effect was found in six and 12 months of follow-up^10^.

A systematic review evaluated orientation groups in populations with chronic diseases such as asthma, hypertension, heart failure, diabetes mellitus, arthritis, among others, but provided little evidence on the achievement of good outcomes in quality of life and health management. The study suggested that more studies should be carried out, especially in order to compare the orientation groups with other strategies^11^.

Thus, this study aimed to evaluate the effect of the orientation group on adherence to treatment and self-care among patients with chronic HF in a specialized clinic.

## Method

This is a randomized controlled trial with parallel-group developed with two groups at the same time, an intervention group (orientation group and nursing consultation) and a control group (nursing consultation).

The study was conducted between October 2012 and February 2014. The inclusion criteria were patients on follow-up in a clinic specialized in heart failure, in the city of Niteroi/RJ, Brazil, who were over 18 years old, diagnosed with HF and included in functional class I to III according to the New York Heart Association (NYHA).

Exclusion criteria were patients with an acute myocardial infarction (AMI) in the three months prior to the study; patients who underwent coronary artery bypass surgery in the month prior to the study or who were indicated for surgery; patients with neurological/cognitive dysfunction; patients who did not live in the cities of Niteroi, São Gonçalo or Rio de Janeiro; and patients who did not have a landline. The intervention occurred during 120 days, with two (02) nursing consultations and sixteen (16) fortnightly group meetings with the intervention group (IG).

The 105 eligible patients were contacted and blindly randomized to the control group and intervention group. Only 56 patients answered the request and then were invited to the study, received orientation and signed a consent form (TCLE) informing on the benefits and risks of the study. At the first visit, the first evaluation of adherence and self-care maintenance, management and confidence was carried out through questionnaires previously adapted and validated for use in Brazil^13^ ^-^ ^14^.

At each nursing visit, a specialist nurse conducted anamnesis, physical examination and evaluation of complementary and laboratory tests. Nursing diagnoses were identified and an educational intervention was conducted through reading of a Guideline on Heart Failure^15^ from the Coração Valente Heart Failure Clinic with the patient and companions. This guideline is used in the conventional follow-up of these patients. In addition, the nursing visit was also used to improve the prescribed treatment.

The intervention “*orientation group*” was based on the classification of Support Group from the *Nursing Intervention Classification* (NIC)^16^. This intervention is defined as “*using a group environment to provide its members with emotional support and health-related information*”.

**Figure 1:**
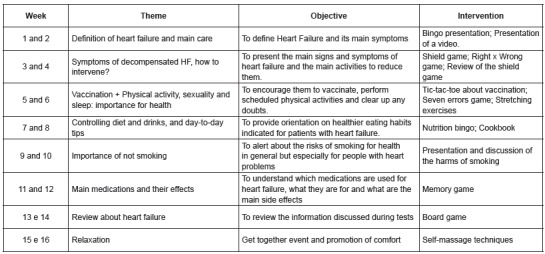
Topics and activities conducted in the meetings of the Orientation Group.

Eight different topics were programmed for the orientation group, based on the recommendations of the Brazilian Guideline for Heart Failure^15^ according to figure 1. The topics were presented with 15 days intervals, however, the same topics were repeated for two consecutive weeks, favoring the participation of the patients. The topics, the objectives and the interventions used are described below. 

The interventions of the Orientation Group were elaborated by the nurses/researchers from the Heart Failure Clinic Coração Valente, with the support of didactic material, such as video, board game, tic-tac-toe, memory game and drawings and paintings. This made the intervention more playful, enjoyable and understandable. 

In addition to the topics previously proposed, the participants of the group had the possibility to express their doubts and report their experiences. The orientation group was open to family members or significant people. Patients who did not participate in at least three meetings with the orientation group were excluded, which totaled one (01) loss per month.

The control group (CG) received the conventional follow-up from the HF Clinic, which consists of nursing, nutritional, physiotherapy and medical consultations. During the nursing consultation, the patients of this group also received an educational intervention based on the Guideline on Heart Failure.

The outcomes assessed in this study were adherence to treatment^17^ and self-care maintenance, management and confidence among patients with HF^10^ in a specialized clinic. Outcomes were assessed at the first visit and reassessed after four months of follow-up.

Adherence to treatment was assessed through a questionnaire with 10 questions, with scores ranging from 0 to 26 points; higher scores indicate better adherence. Adherence is considered adequate when the patient reaches 18 points score, which corresponds to 70%^15^.

Self-care was assessed through a questionnaire with 22 questions divided in three (03) scales: self-care maintenance (10 items), self-care management (6 items) and self-care confidence (6 items)^14^. 

On the self-care maintenance scale, responses range from “never/rarely” to “always/daily”. On the self-care management scale, responses range from “unlikely” to “very likely” and on the self-care confidence they range from “not confident” to “extremely confident”. The questions are about weight and edemas, physical activity, attendance at consultations, diet, medications, signs and symptoms of decompensated HF, management of signs and symptoms, and confidence in decision making^14^.

The scores of each subscale were calculated separately, and each ranged from 0 to 100 points. A self-care score of 70 or more points was considered adequate^14^.

The sample estimate was based on the standard deviation of a previous clinical trial^14^ ^)^ for the same outcomes conducted with HF patients who received nursing interventions. This study set a 95% confidence interval, a margin of error of five percentage points, and power of 80%. Therefore, the inclusion of 20 patients (10 in each group) was estimated. A nine-point difference between groups was considered, based on the outcome of the study in question. The sample calculation was performed using the Winpepi statistical program (v. 14.46). 

The randomization was performed in blocks. A coin flipping determined the allocation of 15 eligible patients in the intervention group or control group. Each patient was identified by an Arabic number on individual cards. After the draw, the numbered cards corresponding to each patient were put in envelopes and stored in a safe place. The person responsible for the randomization was not involved in any other research activity.

During the baseline period, the nurses were blind to the patients’ allocation. The professionals responsible for the randomization, the patient assessment team, and the statistician responsible for data analysis remained blind throughout the study.

The intervention team had no contact with patients in the control group after the baseline period and, therefore, was blind to this group of patients.

The construction and synthesis of the database were done in the software Microsoft Excel (2007) and the analysis was done on the software Statistical Package for the Social Sciences (SPSS), version 20.0.

Categorical variables were expressed through frequency and percentage distributions and the continuous variables through mean and standard deviation. The comparison between the initial and final scores of the intervention and control groups was done through the Student’s t-test. The bivariate p value<0.05 was considered statistically significant for all analyzes.

The study was approved by the Research Ethics Committee of the Antônio Pedro Medical School/ Hospital, under the no. 175.302 and under the registry RBR-7r9f2m in the Brazilian Registry of Clinical Trials.

## Results

Of the 105 patients eligible to participate in the study, fifty-six (56) met the inclusion criteria. Of these, twenty-nine (29) were allocated in the intervention group (IG) and twenty seven (27) in the control group (CG).

During the study, the IG had eighteen (18) losses, seventeen (17) due to attendance to less than three (03) meetings with the orientation group, one (01) for starting dialysis, one (01) for change of address and one (01) for death. Of the twenty-nine (29) patients included, eleven (11) completed the study.

**Figure 2:**
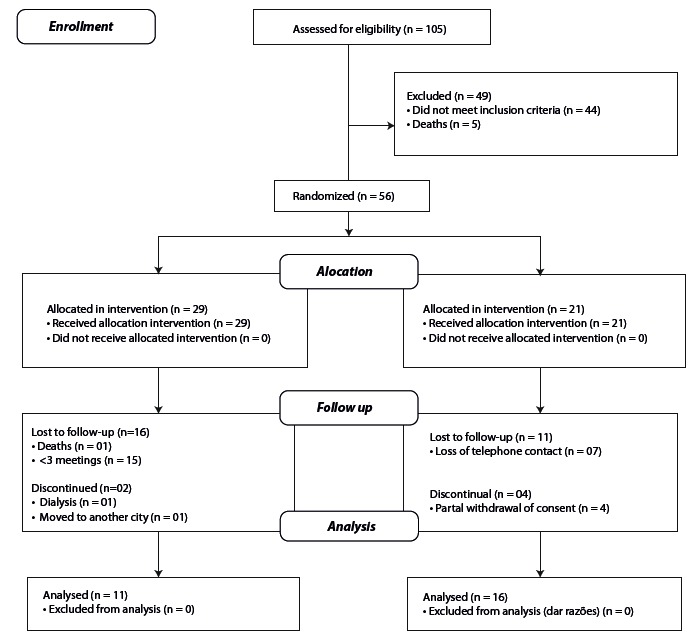
Flowchart of the study Niterói, RJ, 2014

In the CG, 27 patients participated in the first nursing visit. However, there was no possibility of scheduling the second nursing consultation with seven (07) of them, because the phones provided had changed and were unable to receive calls. In addition, four (04) patients refused to continue the study. Thus, in this group, sixteen (16) patients completed the study (figure 2). 

The characteristics of the patients are presented in Table 1. Of the 56 participants, 60.7% were female, married (62.5%), with a mean age of 64.9 ± 12.3 years, most completed elementary education (58.9%), and had a monthly income of R$990.60 (622.00-1200). The duration of the disease was approximately 10.0 ± 4.7 years, and the New York Heart Association class II was prevalent (42.9%). The sample was homogeneous for control group and intervention in practically all variables, only the variable alcoholism presented a statistically significant difference (p=0.041).

**Table 1:** Sociodemographic and clinical characterization of patients with heart failure (n=56). Niteroi, RJ, Brazil, 2014

Variables	Intervention Group (n=29)	Control Group (n=27)	Total (n=56)	p-value
Gender, Female*	16(28.5)	18(32.1)	34(60.7)	0.379^§^
Age^†^	64.1±13.5	65.6±11.8	64.9±12.3	0.463^||^
Skin Color, Parda*	11(19.6)	10(17.9)	21(37.5)	0.379^§^
Occupation, Retired*	20(35.7)	13(23.2)	33(58.9)	0.379^§^
Income^‡^	11100(622.0-1200)	837.5(622-1142)	990.6(622-1200)	0.993^||^
Level of education, Elementary education*	14(25)	19(33.9)	33(58.9)	0.379^§^
Civil status, married*	21(37.5)	14(25)	35(62.5)	0.379^§^
Time from illness onset (years)^†^	9.1±3.4	9.8±5.4	9.4±4.3	0.52^||^
Last hospitalization (months)^‡^	32(0.75-72.7)	5.5(0.75-11.0)	8.0(6.0-11.2)	0.3^¶^
Start of treatment (years)^‡^	6.5(3.7-7.5)	6.5(4.2-8.0)	6.5(4.0-8.0)	0.798^¶^
Left Ventricular Ejection Fraction^†^	61.3±14.0	56.2±14.0	58.5±15.2	0.374^||^
Number of medications used^‡^	6(4-9)	5.5(1.2-4.7)	6(4-7.4)	0.541^¶^
New York Heart Association functional class*	13(23.2)	11(19.6)	24(42.9)	0.379^§^
Smoking*	5(8.9)	3(5.3)	8(14.2)	0.379^§^
Alcoholism*	5(8.9)	11(19.6)	16(28.6)	0.379^§^
Self-care scores				
Maintenance^†^	46.5±13.5	46.3±14.9	45.1±14.2	0.442^||^
Management^‡^	50(32.5-99)	55(40-99)	50(30-99)	0.637^¶^
Confidence^‡^	67(50-83)	61(61-85.4)	61.8±21.7	0.429^¶^
Adhesion score^†^	13.9±3.2	14.4±3.0	14.2±3.1	0.57^||^

* n(%); † mean ± standard deviation; ‡ median (interquartile range25-75); § Chi-square test; || Student t’test; ¶ Mann-Whitney test

The outcomes adherence to treatment and self-care maintenance, management and confidence were evaluated in the initial and final moments of the study, with the scores of the intervention group, the control group and the total sample are presented in Table 2. 

**Table 2:** Mean values of treatment adherence and self-care maintenance, management and confidence at the initial and post-follow-up moments (n=27). Niterói, RJ, Brazil, 2014

Scores	Intervention Group (n=11)		Control Group (n=16)		Total (n=27)		p-value
	Initial	Final	Initial	Final	Initial	Final	Initial
Adherence	13.9±3.6	14.8±2.3	14.2±3.4	14.7±3.5	14.1±3.4	14.7±3.0	0.80
Maintenance	49.1±11.8	49.1±19.7	40.2±17.1	43.7±17.9	43.8±15.5	45.9±18.5	0.15
Management*	56.5±30.7	48.9±18.8	56.1±29.0	27.7±21.4	56.3±29.1	37.2±22.5	0.97
Confidence	74.8±16.2	59.1±14.9	52.6±24.8	67.4±26.1	62.0±23.9	64.0±22.3	0.01

*Student’s t-test/Applied in 20 participants; Intervention Group (9) and Control Group (11).

The initial scores of adherence (14.1 ± 3.4), self-care maintenance (43.8 ± 15.5), self-care management (56.3 ± 29.1) and self-care confidence (62.0 ± 23.9) were lower than the expected for the total population, which was above 18 points for adherence and 70 points on the self-care scales.

There were no significant differences in the outcomes assessed in the initial and final moments between the control and intervention groups. The mean difference obtained at the initial moment and at the end of the follow-up period in the control group and in the intervention group were in adherence (0.47) (p=0.79); maintenance (-3.54) (p=0.54); management (15.73) (p= 0.32) and confidence (-29.38), but the latter was the only statistically significant one (p=0,005), according to Table 3.

**Table 3:** Mean difference scores of adherence to treatment, self-care maintenance, management and confidence (n=27). Niteroi, RJ, Brazil, 2014

Scores	Mean difference (95% CI) Final-Initial	p-value
Adhesion	0.47 (-3.09 - 4.04)	0.79
Maintenance	-3.54 (15.43 - 8.35)	0.54
Management*	15.73 (-16.91 - 48.37)	0.32
Confidence	-29.38 (-47.43 - -11.33)	0.005

* Student’s t-test/Applied in 20 participants; Intervention Group (9) and Control Group (11).

There were no significant differences in the control and intervention groups between the initial and final results. The exception was the self-care confidence score that had a significant negative difference in the intervention group, meaning it was higher in the beginning of the study.

## Discussion

This study evaluated for the first time the effect of an orientation group on the outcomes adherence to treatment and self-care maintenance, management and confidence in patients with chronic HF in a specialized clinic in Brazil. The outcomes were assessed through questionnaires previously translated, adapted and validated for use in Brazil. The results showed that the orientation group was not effective for improving adherence and self-care, and could lead to a reduction in self-care confidence.

The adoption of an educational program cannot guarantee the change of behavior expected in adherence to treatment and in the practice of self-care, considering that what people know and what they actually adopt in their life is often different. Adherence to treatment is often affected by reasons related to the HF itself and its comorbidities; in addition, internal and external factors to the individual such as motivation, ability to understand, existence of adequate health services, and other factors directly influence this aspect^18^ ^-^ ^19^.

The initial scores of the sample were low in adherence (14.11 ± 3.41), self-care maintenance (43.82 ± 15.54), self-care management (56.26 ± 29.10) and self-care confidence (62.01 ± 23.94). Using as evaluation methods two scales used worldwide for self-care assessment, the Self-care Heart Failure Index and the European Heart Failure Self-Care, a comparison between self-care in 15 countries showed better indexes in developed countries^9^ ^,^ ^20^ ^-^ ^21^.

In a study with 197 Americans with HF, 56.1% with NYHA III, the self-care maintenance score was (70.5 ± 14.3), the self-care management score was (65.4 ± 22.4) and self-care confidence score was (70.2 ± 16.6)^22^. All these values are higher than we found in this study. The education of the patient related to better educational and socioeconomic conditions, may be the explanation for the difference between these scores, since in this study more than 80% of the interviewees had only elementary education I and II.

In the Chinese population, with a population of 182 patients with HF, from three hospitals, 71% men and 79% with NYHA II/III, the scores were: self-care maintenance (43.5 ± 16.7), self-care management (51.4 ± 21.6) and self-care confidence (52.0 ± 21.1);. They had self-care maintenance scores similar to this study and lower confidence score^21^. 

The comprehension of the orientation provided may vary depending on the level of education, and the intervention should be adapted to this condition. In addition, access to the medication prescribed may be impaired, since it may be difficult for patients to correctly understand what is prescribed in order to acquire these medications^18^. Therefore, low education level and income are predictors for low adherence to treatment and self-care, leading to decompensated HF and increasing hospital readmissions^23^.

In this study, the strategies used in the orientation group were based on recreational activities, such as movies and games, to facilitate the understanding of the content discussed. The multi-professional team supported the development of the activities proposed. Considering that social support is an important element for adherence and self-care, the orientation group was open to family members and had the participation of spouses, siblings and children of the participants.

Social support was also offered in nursing consultations, which may explain the improvement of self-care confidence in the control group, which had an initial score of 52.63 ± 24.76 and a final score of 67.41 ± 26.15.

On the other hand, the negative change in the self-care confidence score in the IG can be attributed to the knowledge acquired during the orientation group meetings. Perhaps, the comprehension of the condition, the stage of the disease, its incurability, and the difficulties experienced by several patients may amplify the patients’ perception and make them feel insecure to state that they are extremely confident in these issues.

People normally can’t judge themselves, so they usually feel capable and confident to carry out tasks. Unaware of their ignorance, they overestimate their experience and talent, and consider themselves competent^24^. This overestimation is more frequent among the less capable, since the more competent can recognize their true level of abilities and compare it with others^5^.

Thus, before the intervention group, the participants were more confident. As they became more competent to evaluate their health status and self-care, their self-report was more real, which may explain their lower level of self-care confidence.

It is also important to understand the full scope of the concepts of adherence and self-care. These matters go beyond monitoring of signs and symptoms, and should include issues such as comfort, environment, emotional factors, support system such as family and friends, basic hygiene, sleep and rest, eating habits, daily activities, work, leisure and ability to communicate and interact with the world^25^.

The concepts of adherence and self-care, therefore, are complex. They involve logical thinking in order to assess health condition and promote behavior changes, which are difficult to measure. Therefore, in order to find changes that have clinical impact, it may be necessary to extend the evaluation for a longer follow-up period.

The main limitation of the study was the low attendance to the Orientation Group, which hindered the follow-up and the evaluation of the outcomes. The authors attributed this limitation to the difficulty of public transportation to the specialized clinic, since it is located in an urban area, with a lot of traffic and it is difficult for people with special needs to access the location. It is worth mentioning that patients with HF may be intolerant to some efforts, such as ramps and ladders. The fortnightly meetings were also an increase in financial expenses, so despite their acceptance to participate in the study and their positive verbal reports regarding the activities carried out in the group, some participants could not attend regularly. 

Thus, the irregularity in attendance at the meetings obstructed the interaction between professionals, patients and family members, hampering the creation of a nurse-patient bond, essential to the activity of the intervention group. This may also have been a limitation to the intervention’s success. Finally, with the easy access to cell phone lines, it is easier to change numbers and carriers, making it more difficult to find the patients. 

A study that aimed to discover why patients with HF participated or not in a self-care support program found that the main reasons for permanence in these programs were support, friendship, exchanging information, acceptance and control of the disease, exchange of experiences and combating depression. However, the most relevant causes for abandonment were the physical impairment imposed by illness, depression, uninteresting educational materials, and very small groups or individual strategies^26^.

Thus, the results found should be analysed with caution, and multicenter studies with longer follow-up periods should be conducted in order to obtain a better evaluation of the orientation group and its effect on adherence and self-care. However, this study was relevant, since, in a controlled manner, through a randomized controlled trial, it evidenced the difficulties of investigating the effect of group interventions for patients with HF.

A 6-month home and telephone follow-up study demonstrated a 27% relative reduction in the outcomes hospitalization, emergency care, or death. The study also improved HF knowledge and self-care actions^27^.

Thus, longer follow-up periods with combined strategies seem to be better for promoting adherence and enhancing self-care for this type of patients. Therefore, this may be an alternative to improve these outcomes among individuals in support groups.

## Conclusion

The Orientation Group did not alter adherence and self-care among patients with HF in a specialized clinic. However, it is an important educational strategy in the healthcare area, specifically for chronic patients, and it should be better explored in other settings and with other strategies.

Patient confidence in their self-care decreased with the group intervention; however, this result can be considered positive in medium and long term, since it can spark an interest in understanding their health condition and the treatment. Maintaining the nursing intervention and associating it with the support of psychologists, social workers and occupational therapists may be an alternative to solve the problem. 

Therefore, this study is significant because it demonstrated that well-structured strategies may not achieve the expected goal, but can provide evidence for the construction of more effective interventions. 

Multicenter studies with larger samples and in medium- and long-term may produce positive results in the outcomes adherence and self-care through group interventions.
